# Foreign Summary

**Published:** 1839-04-06

**Authors:** 


### FOREIGN SUMMARY.

*Swallowing Pins and Needles.*—A very curious medico-legal fact is narrated in the *Gazette des Tribunaux*, and the *Droit* of the 16th November. A servant girl, of seventeen, named Rose Melanie Selter, was tried before the Court of Assize of the Seine, for attempting to kill a child, aged two months and a half, by making it swallow ten pins. The case alleged was as follows:—

The Sieur and Dame Fournereau have an only child, now about five months old.* It was suckled by its mother, and was in the finest health, when, on the 7th of last April, it was attacked with dyspnœa and fits of suffocation, which made its parents fear for its life. On the following days its sufferings continued, and it seemed as if there was something in the infant's stomach and throat which obstructed respiration. However, on the 10th of April the pains ceased, and the infant recovered its health.

The cause of the attack was unknown, until, on the morning of the 11th of April, Fournereau's wife found three pins in the child's stool, four more in the evening, and three the next morning; making a sum total of ten pins that the child had swallowed. Fournereau and his wife attributed what had happened to the malice of the servant girl, and discharged her.

On their complaint, Selter was taken up, and confessed before the commissary of police that she had made the child swallow ten pins on the 7th and 8th of April; and that she had done so in order to get herself discharged and sent home to her parents, who forced her to go to service. In the written statement, the prisoner still said that she had made the child swallow the pins, but asserted that it was all done on one occasion; and that she must have lost her reason to do such a thing, for she loved the child, and had no cause for animosity against her master and mistress. She also alleged that at certain periods she was worried by her blood to such a degree that she did not know what she was about.

It seems that three or four years ago the prisoner had some symptoms of insanity, consisting of a nervous agitation, which made her run about the country without any object, and compelled the Sieur Maugin to send her home to her father.

A physician was ordered to visit her in prison, and make his observations upon her for a certain time. He reported that no symptom of derangement had appeared since her imprisonment. Dr. Ollivier, of Angers, being consulted by the court as to the seriousness of the attempt, medically considered, replied as follows:—

"The introduction of the pins into the child's body did not produce any serious symptom; this is not surprising, for there are numerous examples of the same kind on record. Thus there is a case of a young girl who had swallowed pins in her childhood, and did not get rid of them till fifteen years afterwards. There are pains, indeed, and a feeling of suffocation at the moment of their passing into the œsophagus, and that is all. A young girl who was insane, a toy and doll maker by trade, and who also had pins about her, swallowed fourteen hundred of them, which were all found in her body; her muscles were as thickly set with them as so many pincushions. Nevertheless, her death was quite independent of this occurrence. Hence, the rule is, that bad symptoms are not produced, but there is a considerable number of exceptions, where abscesses

* The indictment having been drawn up in June, as we suppose.—*Translator.*

in the liver of the abdomen, and death itself, were caused by pins.

"The story which the prisoner first told me is possible, and the pins may all have been given to the child at once. As to whether they were swallowed head or point foremost, I cannot answer that question; for though they may have been passed with the head foremost, they need not have been introduced in the same way, since they may have been reversed in their passage.

"It was next my duty to examine the state of the prisoner, and to do this effectually, I inquired into her previous history. After having lived in Paris from her earliest years, she passed a year and half in her native district. The official papers contain the notes and descriptions of persons who saw her during that period. I was struck with the contrast between the physical development of the girl and her slender intelligence. She is sixteen and a half, and you would have taken her to be twenty; but though physically developed, her conduct is that of a child. I have observed alternations of good and bad health since her confinement in prison. She suffers from headache very frequently; she feels very drowsy, and it is paticularly at the catamenial periods that she is in this state.

"Selter told me at first that it was at one of these periods that she committed the crime of which she is accused. It was my duty to draw conclusions from all these facts, and I must say that nothing, either in the conduct or the answers of the prisoner, showed any disorder of the intellectual faculties. Nevertheless, after having maturely considered the interesting medico-legal questions which arise in this case, I declare, that when I connect together the habits of the prisoner's childhood with what is extraordinary and motiveless in the act of which she is accused, I have my doubts. (A sensation in court.) This uncertainty is increased when we think of the temporary disturbance which certain periods that I have just mentioned cause in woman. It is my duty to tell you, that I have my doubts. I do not now oppose what I said in my report, but I am less decided than I was."

The Advocate-General.—"There is a fact that you do not know, because it has only come out in the proceedings. The prisoner did not show the least emotion during the whole course of the child's illness. What deduction can you draw from this?"

Dr. Ollivier.—"To a certain extent this would seem to confirm what I have just said. If she had had sensibility, as every one else has, she would not have been able to see the child's sufferings without betraying herself by her uneasiness. It may be possible that she acted without intention, mechanically, and by one of those instinctive impulses of which we may each find examples by examining ourselves."

After a discussion between the advocate-general and Dr. Ollivier on the state of the prisoner's intellect, and hearing some witnesses for the defence, M. Plougoulm, the advocate-general, rose, and gave up the prosecution.

After a short deliberation, the jury brought in a verdict of not guilty, and the president declared the prisoner acquitted. The girl seemed unconscious of what was going on; she heard her acquittal without betraying the least emotion, and did not think of leaving the court till told to do so by the *gendarmes*.

The preceding facts sufficiently prove that there was no *malice prepense* in the prisoner when she made the child swallow the pins, and that the act can in strictness be called nothing but folly or madness; and the judgment of the court was founded on this supposition.

The following is the medico-legal question arising from this case. Dupuytren expresses himself on this subject as follows:—

"I have seen at the Hôtel Dieu, a considerable number of women and children afflicted with this mania, and suffering under the same symptoms. The most remarkable of these cases was one of a woman, who, in consequence of swallowing an incredible number of pins and needles, had become frightfully thin, and was obliged to keep quiet still in bed, from the acute pain which was caused, on the slightest motion, by the needles and pins, which made their way out from every part of her skin. I opened more than a hundred collections of pus in this woman, at the bottom of which I always found one or two needles or pins. On the surface of this unfortunate person's body there were always fifty or sixty abscesses or tumours caused by the presence of as many of these foreign bodies; which, when added to the number of those which nature was not yet strong enough to drive towards the skin, formed a fearful sum total. It is easy to see that if the presence of a single one of these foreign bodies makes motion difficult and painful, so great a number must bring on a general debility, continued fever, and fatal marasmus: and, in fact, the woman of whom I am speaking died in a hectic state. When her body was opened, several hundred pins and needles were found spread throughout the various organs, the limbs, the cellular tissue, and the muscles; in short, in every part of the body." (Dupuytren, *Blessures par armes de guerie*, t. ier, p. 82.)

We see that Dupuytren is far from thinking that a great number of needles or pins can be swallowed harmlessly. Yet there are facts which prove, as Dr. Ollivier says, that these pointed bodies may pass from the alimentary canal into the neighbouring organs by gently penetrating the tissues, and at length creeping towards the surface of the body without causing any serious symptoms. Every one knows, for instance, the history of the girl at Copenhagen, who had a passion for swallowing needles, and in whom a number of points were observed in the skin, giving exit to these instruments.

In other instances these bodies become enveloped in mucus, slip into the bowels, and make their way out by the anus, as in the case of Fournereau.

"Foreign bodies," says Boyer, "when long, thin, and pointed, such as needles and pins, sometimes traverse the stomach or intestines, and reach the liver or mesentery. But most frequently they pass without causing pain or inflammation, and appear under the skin in parts more or less distant from the alimentary passages. Lastly, foreign bodies have been known to traverse the intestines, enter the bladder, and pass out of the urethra with the urine."—Boyer, *Malad. chir.* t. vii. p. 198.

Still more surprising cases might be cited:—"An officer afflicted with a suicidal mania pushed one of those long black pins, known under the name of curling pins, into the region of the heart. It penetrated the pericardium, reached the heart, and remained there, without causing any symptoms, during a period which could not be ascertained. There was nothing to indicate its presence during life. The pin was not found till after the death of the officer, which he inflicted on himself in a different way."—(Dupuytren, *op. cit.* p. 79.)

Although it is true that the introduction of needles and pins is not always followed by serious symptoms, yet we must not make this a general rule. Science has not hitherto afforded a sufficient number of facts to allow us to decide the question so absolutely as Dr. Ollivier has thought himself justified in doing; we do not blame his judgment in the case above given, where we are entirely of his opinion, but we do not side with him as to the general harmlessness of the accident in question. We have just seen that Dupuytren thought it a very serious one, and his opinion was based upon experience.

When a needle or pin sticks in the upper part of the œsophagus, or in the pharynx, it may cause violent symptoms of suffocation. We once saw a woman who fell into frightful convulsions from having pushed a needle, which was accidentally contained in the bread she was eating, into the velum palati. In the Memoirs of the Academy of Surgery there is a case of suffocation occurring in a child from the presence of a pin which had pierced the larynx transversely. Fabricius Hildanus saw death occur in consequence of a small body, pointed like a pin, sticking in the œsophagus. During deglutition, therefore, pins and needles may stick in the upper part of the pharynx, and cause serious symptoms. We once saw a young and robust man, who died at the Hôtel Dieu, under Dupuytren's care, in consequence of an abscess caused by a bone sticking in the pharynx.

It follows from the preceding facts, that when one or more pins have been swallowed, we can never tell *a priori* what will be the consequence; there are examples both of recovery and of death.

Let us now suppose that a physician is called in the moment the accident has happened, or soon after, the needles or pins being in the stomach; what ought he to do? Hear Portal:—

"I saw a young man, who, during a drinking bout, challenged his companions to swallow a part of his glass; he broke the fragments of his glass with his teeth, and then swallowed them; but not with impunity, He was soon seized with frightful cardialgia; convulsive movements came on, and fears were entertained for the life of this giddy-headed young fellow, when his friends came for me. I first had him bled; but as the principal object of the treatment was to extract the glass which caused the symptoms, I was much embarassed as to the means of doing so. On the one hand, I saw that tartar emetic would increase the irritation and contraction of the stomach, and that the glass would get more closely into its parietes; on the other hand, purgatives would drive the glass into the intestinal canal, the long extended surfaces of which would probably become excoriated. I thought it right, therefore, to advise the patient to fill his stomach with some food which might serve as a recipient to the glass, and then to produce vomiting. Some cabbages were procured and boiled; the patient ate a considerable quantity of them, and I then gave him two grains of tartar emetic in a glass of water. The patient soon vomited, and threw up a considerable quantity of glass among the cabbage. He subsequently took a good deal of milk, was put into a bath, and had some emollient clysters; and as he had become very lean, in spite of these methodical aids, I advised him to drink asses' milk, which he did for more than a month, and which restored him to his former state of health."—*Lon. Med. Gaz. from Gazette des Hopitaux*, November, 1838.

---

*On the formation of Moulding Tablets for Fractures, &c.* By Alfred Smee.—The importance of a substance that can be moulded accurately to any part of the body at a moment's notice, must be admitted by every member of the medical profession; yet many difficulties attend the formation of a composition which shall, at the period of its application, be so yielding and soft, that it may take an accurate cast of any part, and when dry, shall still retain the form given to it, and become sufficiently hard to resist external impressions, and at the same time shall be tough, elastic, and devoid of brittleness and much flexibility; and further difficulties present themselves where the capability of its being quickly dried is required. The advantage of lightness and cheapness is also a great desideratum.

As I had frequently noticed that the composition of gum arabic and whiting, when dry, possessed great hardness and toughness, and yet was so free from brittleness that it could scarcely be pounded in a mortar, I was determined to ascertain how far it would answer to make tablets, which might be used to form extemporaneous splints.

For this purpose a piece of coarse sheeting was copiously brushed over on one surface with a thick solution of gum, after which it was covered with a composition made by rubbing whiting with mucilage, continually adding the powder until the whole was of the consistence of thick paste: a second piece of sheeting was now rubbed over on one side with the solution of gum, and the moistened side applied upon the composition with which the piece of sheeting had been covered, and we thus had two thicknesses of sheeting with an intervening layer of the composition of mucilage and whiting, the thickness of which may be increased or diminished as strength or lightness is desired. The whole was then dried, and formed a tablet about the thickness of slight pasteboard.

This experiment succeeded beyond my most sanguine expectations; for, whilst the tablet remained dry, it was exceedingly hard, and, when sponged over with a little warm water, became so yielding, that, by moulding it with the fingers, a cast could be taken of any part of the body. The hand and knuckles were defined with great accuracy, and I succeeded, by a little management, in taking a cast of the greater part of the face. It is sometimes advisable not to allow the substance to dry upon the part on which it is moulded; but after the depressions and elevations have been traced with the fingers, it should be carefully removed and partially dried before the fire; and as soon as the texture is sufficiently dry to retain its shape, it may be placed near a stone, or even on the hob of a grate, without fear of corrugating or becoming otherwise deformed. In most cases, however, this drying is quite unnecessary, it being requisite only to envelope the moist tablet with a bandage. A cast thus taken is extremely hard and tenacious, so that when not much thicker than a wafer, it may be struck violently and repeatedly against any hard substance, and not be destroyed. It possessed but slight flexibility, and, after having been bent, it returned to its previous form, showing considerable elasticity. It was neither liable to be torn nor broken; and lastly, it possessed the advantage of lightness combined with durability. Whilst in search of a moulding substance I thought it advisable to try various compositions, in order that the best might be selected, but none appeared so excellent as that last described.

A composition of powdered starch and gum was spread upon linen wetted with the solution, as in the first instance, when it afforded a good and firm tablet, but perhaps not equal to the first. A paste made with gum and flour formed a good composition; but in all cases where flour is used there is a liability to more or less contraction. A mixture of plaster of Paris and gum dried very speedily, but was apt to crack, and did not wear well.

Compositions of white of egg, with the same substances to thicken it as were used to thicken the solution of gum, were next tried, the linen cloth being first smeared over with the albumen; but none were found to answer; and it was singular that the mixture of the sulphate of lime and the white of egg had so little firmness that it fell to powder when dried.

Boiled glue and whiting formed a hard tablet, inferior, though slightly cheaper than that first described; and its unpleasant smell would prevent its use, except in hospital or military practice.

Similar compositions of flour paste were found utterly useless, having neither consistence, cohesion, nor strength. The decoctions of the Iceland moss and linseed were found also inapplicable. The preparations of dextrine were next tried, and a mixture of carbonate of lime with the solution of dextrine made a composition which answered very well.

Of all these preparations—and many others that were tried—few were applicable, and none in all respects equal to the composition of gum and whiting, both of which substances are always easily obtained, and have the additional advantage of cheapness. The solution of gum which was found most adapted contained 10 or 12 oz. of gum to the pint of water.

As far as regards the nature and texture of the cloth, it is to be remarked that linen is stronger than cotton, and less liable to be torn, and therefore to be preferred. Of the various kinds of linen, none moulds so perfectly as moderately coarse old sheeting; for when the tablets were made of finer Irish, they were very inferior in this respect.

The application of these tablets is rather extensive; they may be used with great advantage for all fractures of the metacarpal bones; also for those of the forearm, or even for the humerus.

When the humerus is fractured, the method which has been adopted is to cut a piece of paper somewhat into the shape of the required splint. It should cover a portion of the pectoralis major, and extend as high as the bend of the neck, and include the whole of the scapula. From this broad plate a piece decends to the bend of the elbow, and should be sufficiently wide to cover about two-thirds of the outer part of the arm. The paper is then placed on one of the prepared tablets, which is cut to a similar shape. The piece thus prepared is moistened until it becomes perfectly soft, and it is then moulded on the arm and neck. From the general shape of these parts, there will be found a superfluity of substance about the deltoid which must be pinched up and turned down, so as to form a fold over the other part. The splint then may be in a degree dried, and its inner surface lined with lint. The whole is to be enveloped with a starched roller.*

This mode of proceeding may appear tedious, but it is a source of much comfort to the patient; for whilst the upper arm is enveloped in this hard case, so that it is quite immoveable, the forearm and hand may be left loose, and the patient may in some degree enjoy the use of them. The benefit of this mode of treating fractures is not confined to the patient only: it lessens also the labour of the surgeon; for when the injured limb is once put up in this manner, it requires no further attention for days, weeks, or even till the cure is accomplished.

Its application to chronic diseases of the joints will be found particularly useful. In these cases, two lateral splints are to be formed, and envelop-

*The roller is merely soaked in boiled starch, and wound up in the usual manner, before it is applied.

ed in a starch roller. It is hardly necessary to add, that in fractures of the lower jaw it must prove a valuable auxiliary. Great, however, as these advantages may be, perhaps they are trifling in comparison with the importance of its application to simple fractures of the leg. The mode of treating these fractures at St. Bartholomew's Hospital has been for some months the method first adopted by Mr. John Lawrence, of Brighton. His plan was to form two strong splints on either side of the injured leg, by successive layers of pieces of bandage united together by white of egg and flour. Now as far as this method is concerned, it requires no improvement, as durability, strength, and an accurate cast, are obtained by this mode of proceeding, and the numerous cases which have been treated by it at the hospital show its complete success. By using the tablets formed of gum and whiting upon the same plan as that of Mr. John Lawrence, a great saving of the surgeon's time is effected, and equal firmness and durability obtained.

The mode in which I have made splints for the leg, is first to obtain the exact shape by drawing a piece of sheeting or paper round the leg, and marking the part which corresponds to the tibia for the whole length of the leg, and continuing the line on the foot to the extent that it may be considered necessary to cover.* By this means, it is apparent that the exact size of the limb is obtained; but as the leg is to be enclosed by two splints, it becomes necessary to divide the cloth into two, which will give an exact pattern of either splint. These splints are to be moistened and moulded, and after being first lined with lint, or leather, the whole is to be enveloped by a roller soaked in boiled starch.

This composition of gum and whiting has answered perfectly in all the cases in which it has been tried, and splints made with it are perhaps superior to the splints made with flour and white of egg; because, when dry, they preserve accurately the shape of the limb, and do not at all corrugate, which all compositions of flour are liable at times to do.

Fractures of the patella are treated in a similar way, a splint being placed on either side of the knee, extending from about the centre of the thigh to about the centre of the leg. The patella is not to be covered with these splints, but a gap corresponding to its shape left, and the two pieces of splints are not to meet accurately at any part, but an interval is to be left of about 3–4ths of an inch, or an inch, during their whole extent.

In enveloping these splints for fractures, they are not to be applied when there is much inflammation or swelling, but the part should be allowed first to get into a perfectly quiet state. Leeches, cold water, or poultices, should be applied, if necessary, to effect this object. In general, a delay of a week, ten days, or even sometimes three weeks, is required; but in some favourable cases there is no occasion to wait, and the splints may be applied with safety and advantage on the second or third day after the accident.

This mode has also been adopted in favourable cases of compound fracture, but most surgeons are agreed never to cover these wounds with concealing bandages.

It is not for me to expatiate upon the advantages with which this method of treating fractures is attended, for that belongs rather to Mr. John Lawrence, as the first adopter of the principle; but the fixing of the bones more firmly and securely than can be accomplished by any other method—the prevention of loss of health, by enabling the patients to walk on the fourth or fifth day after receiving the accident, and permitting them to be removed to a situation more healthy and airy, the prevention of stiff joints and the more speedy and final uniting of the bone—are advantages too great to be passed over unmentioned. These advantages are likely to be enjoyed by a greater number when the time required for the first application of the splints is diminished, and the objection is removed of allowing the limb to remain without bandages during the time required for the drying of the splints: the tablets which I have described possess these additional advantages, and with them superior cheapness is also conjoined.—*Lon. Med. Gaz.*

*Either splint should overlap the heel and under surface of the foot in cases where they are used immediately after the accident; but where their application is delayed, this is of no importance.

---

*Exostosis of the Pelvis of unusually rapid growth.* By William Lawrence.—Mary Petit, 30 years of age, has gained her livelihood by selling fruit in the streets, and has led an intemperate life. About six weeks before she came to the hospital she observed that the veins of the right leg were swollen, and she attributed the circumstance to over exertion. Soon after, a tumour, the size of a nut, appeared in the situation of the femoral absorbent glands on the same side; it did not prevent her from following her occupation. As the swelling increased, and became painful, especially on exertion, she applied at the hospital, and was admitted on December 21, 1837. At this time the veins of the right lower extremity were varicose in a slight degree, and there was a tumour in the bend of the thigh not larger than a pullet's egg. Being of oval figure, with slight irregularities of surface, it it was considered to be an enlargement of the femoral glands. It was free from redness, and not painful on pressure; yet the patient complained of considerable uneasiness in the part.

The Ung. Potassæ Hydriodatis to be rubbed on the swelling,

29th.—Great pain in the swelling.

Ten leeches; linseed poultice.

Jan. 1st, 1838.—The tumor is larger, and so painful as to prevent rest at night. The limb is œdematous.

Four grains of Potassæ Hydriod. in two ounces of Decoct. Sarsap. Co. three times daily. One-third of a grain of Muriate of Morphine every night.

9th.—The limb more swelled, with increase of pain.

The dose of Morph. Mur. increased to half a grain. An ointment consisting, of Cerat. Cetac. ℥ss., with Pulv. Opii, ʒj. to be rubbed on the swelling in the thigh night and morning.

The tumour increased rapidly, and became more and more painful. Having been at first moveable, like a glandular swelling it became fixed, and extended along the inside of the thigh, in the direction of the pubes and ischium, forming a large mass, of firm feel, not painful on pressure, filling up the space between the pelvis and thigh. In the early part of April the growth was found to extend behind the abdominal muscles, towards the cavity of the pelvis. It continued to increase rapidly, both on the outside and inside of that cavity, its growth being attended with correspondent general swelling of the limb.

On May 1st, the tumour, which is hard and incompressible, has stretched across the pelvis to the left side of the body; and the left leg begins to swell. On May 17th, it had mearly reached the umbilicus. Her sufferings were constant and acute, and only imperfectly removed by opiates; her strength was thus exhausted, and dyspnœa came on in June, when she was so reduced and enfeebled, that death was expected daily. She lingered till July 1st.

Neither local nor general means had the slightest effect on the complaint. The treatment consisted in the free uses of opiates, particularly of the muriate of morphine, and in the allowance of such nutritious diet and cordials, including animal food, sago, porter, and wine, as the weakness required, and the appetite would admit of.

The disease consisted of an enormous mass growing from both sides of the pubes and ischium, extending downwards to the groin and inside of the thigh upwards to the pelvis and abdomen. The viscera were necessarily displaced, the bladder and internal organs of generation being pushed towards the left side; while the abdominal contents were thrust upwards against the diaphragm. The basis and centre of the mass were firm bone, and the growth at its origin was identified with the bone from which it proceeded. The exterior was of a softer composition, and displayed a fibrous texture more or less firm. On the surface this exhibited, in some situations, cells containing either serous fluid or grumous blood.

No disease was observed in the absorbent glands.

The thoracic viscera were healthy.

In its origin, and in the composition of its basis and interior, this tumour was an exostosis; in the rapidity of its growth, in the severe pain which accompanied it, and in the constitution of its exterior, the characters were those of a malignant growth. I have seen a somewhat similar combination of the bony excrescence, with softer growth, the latter being in some parts of nearly medullary consistence, and formed into cells containing bloody fluid, in the tibia, where, however, the disease was of long standing. The limb was amputated, and there was no reproduction of disease. Had the disease been seated in the tibia in the present instance, it would have been right to amputate.—*Ib.*

---

*State of Medicine in Algiers.*—Dr. A. Von Schönberg, chief physician to the king of Denmark, took part in the expedition against Algiers, and communicated the result of his observations to the Royal Medical Society at Copenhagen. He also published them in a separate form, and the following abridgment of his work is from a review of his work in the *Zeitschrift für die gesammte Medicine*, for January, 1838:—

The climate of Algiers is, upon the whole, healthy, though the air is polluted by putrescent matter. The highest temperature observed by Dr. Schönberg was 90½° of Fahrenheit; the lowest, 27½°.

In general, the character of the diseases in spring (when the blood, as the natives say, is heated) is inflammatory; in summer, it is gastric and bilious, without any disposition to pass into a nervous type, unless treated with violent medicines. In autumn, inflammations again prevail; and in winter, rheumatisms and catarrhs. Intermittent fevers are occasionally seen in the neighbourhood, as well as putrid and marsh fevers near morasses.

The thoracic organs are not so often affected as those of the abdomen; scarcely two persons out of a hundred are free from piles; diseases of the spleen and liver, cachexia, and dropsies, are frequent. Pulmonary phthisis is not rare, commonly following a neglected catarrh. The Bedouin attendants at the baths suffer the most [from phthisis ?], and cauterize their limbs, particularly at the wrist and elbow, with burning wood. The juices of plants, and balsams, are the ordinary remedies; if in six months they do no good, the case passes for mortal.

The climate seems to predispose persons to nervous diseases; there are a number of insane and epileptic persons, and a peculiar kind of headache is found here. Tetanus and locked-jaw often arise without any wound, and, indeed, without any cause that can be discovered. Purges given till they have slightly weakened the patient, then opium in enormous doses, and, above all, musk, form the treatment which is relatively the most successful. The plague is not endemic, but is brought in from the Levant: it then rages violently, though with remissions, and one sees people die of it in the streets.

Venereal diseases, especially of the skin, are much diffused, perhaps through the frequent use of vapour-baths, and do not hinder the patients from going into company. Sarsaparilla in powder, and given in decoction for a beverage, is administered for forty days, and this, with a diet confined to bread and raisins, and the use of baths, forms the course; this is sometimes begun over again, and is then backed with calomel, given according to the fancy of the prescriber. It is easy, therefore, to understand that salivation must be common, to the terror of the physicians and the patients. Buboes are treated by touching the circumference with the actual cautery.

The feast of Bagram gives rise to a colic which occurs in every house; for after a fast of thirty days the Algerines indemnify themselves by a feast lasting three days, where the favourite articles are pastry and sweet things. This colic is frightful from its treatment: two scarfs are put round the neck of the patient, and twisted together, till he seems strangled; they are then loosened, and the patient, still quite stupefied, is violently pushed backwards and forwards by the knees of two men standing opposite to each other.

Dropsies are not rare, and among them hydroceles of great size are the most frequent. It is certain that the Arabs who live in the mountains possess very efficacious secret remedies against stone, gout, and rheumatism. A violent diarrhœa, with tenesmus, was soon cured by burnt cork in rum, which is also employed in Malta.

In a case of headache, a salivation was beneficially produced by friction under the tongue with a piece of woollen cloth, and then sprinkling the tumour thus excited with salt and onions.

The French gave the name of epidemic dysentery to the disease which caused the greatest havoc in their army. Dr. Schönberg prefers calling it a cholera. It is an ulceration of the colon, unaccompanied by typhus fever, chronic in duration, and curable by leeches and emulsions. The headache above mentioned is an intermittent hemicrania, which attacks foreigners in particular. An Italian, who had suffered eight years under this disease, which even deprived him of consciousness, got well with ipecacuanha and quinine; and so did another. In one whole family, consisting of parents and children, the attack began with amblyopia, which increased to total blindness, lasting several hours. One member of the family recovered on leaving Algiers, and was again attacked on his return.

Diseases of the eye are all treated with lotions, and injurious ones too, as appears from the great number of the blind. Egyptian ophthalmia is not so common as at Tripoli.

Fractures are cured by covering them with gypsum, or by amputation. The limb is cut off by a blow of a sword, and then the stump is dipped in boiling pitch, or touched with a red-hot iron. The actual cautery is also used in internal complaints, such as colic; and in cases of incarcerated hernia, what they call bleeding consists in scarifying the forehead and temples with a razor, by which the temporal artery is sometimes injured, or in wounding the Schneiderian membrane with a pointed bit of wood Accupuncturation of the temples is much used against headache.

Burns and ulcers are cauterized, and then very successfully treated with the powder of a certain plant.

A tumour in the antrum of Highmore is of frequent occurrence; it is painful, becomes covered as if with a syphilitic herpes, and sooner or later involves the bones. For the sting of the scorpion, (an animal which injures only when opposed, and is not common here,) the animal itself is crushed and put upon the spot: amulets, as may be supposed, are used against every disorder.

Dr. Bohn first introduced vaccination, and practised it in the family of the deposed Dey himself, who, however, did not give him a princely fee; and, generally speaking, people are here unwilling to give aught to physician or apothecary.

As to the native physicians, the Dey had a kind of protomedicus, who decided medico-legal questions, and created other physicians for a few piastres, without being exactly able to read and write. If a man was able to shave well, if he could compound a plaster, and cure a hurt, he bought the privilege, and prescribed at his own pleasure the whole contents of any of the six Moorish apothecaries' shops; bark with or without theriaca at all times; and in all fevers, opium, sarsaparilla, calomel, pimento, cantharides, and opodeldoc. Ismael Ben Mehmed enjoyed the greatest share of public confidence; he gave Dr. Schönberg an extract from the Arabic work of Ben Huesina, who lived seven hundred years ago, and a catalogue of his own drugs. His shop, the largest in the town, contained seventy jars, thirty bottles, twenty boxes, and several drawers. He obtained medicines from abroad, prepared others himself, and possesses a still and retort. He is afraid of mercury against syphilis, and thinks he can do without it.

Ismael Ben Mehmed is acquainted with remittent and intermittent fevers, and their varieties. His surgical apparatus consisted of a common case of dressing instruments.

Midwifery may be practised by any woman, but there is a certain experience which is handed down as an inheritance. There are both Moorish and Jewish midwifes, and several of the latter are in repute. One of them attends from one hundred and thirty to one hundred and fifty labours yearly, and she says that from three to five in a hundred require artificial aid, chiefly from the presentation of the arm or breech. The labour seldom lasts two days, and it is rare that the new-born infant or a woman in child-bed dies. Puerperal fever is unknown, and milk fever is mild.

The arrangement of the baths in Algiers is peculiar. Sea-bathing is used only for negro slaves; when they have the itch, they are driven into the sea once or twice a day, till they recover. Sweating baths are the usual ones. The bathing-house has a cupola like a mosque, and exhibits the most curious mixture of magnificence and poverty, of cleanliness and dirt. It consists of two large rooms; in the first there are galleries covered with mats for undressing and reposing; in the bathing-room, which is much larger, are the pipes. As soon as the bather begins to perspire, he is laid down by the attendants, and rubbed, first with the bare hand, then with a woollen glove, afterwards with a lather of soap, while merry songs are sung, and lastly, washed with cold water. When the perspiration is over, the attendants again stretch and pull all the limbs of the bather, to make them supple.

A mineral bath in the neighbourhood of Algiers, has come more and more into fashion since the nearly perfect recovery of the first minister from lepra, a disease which is by no means uncommon.

There is nothing which can be called a police for public health. The rubbish from trades of all kinds, especially oil manufactories, slaughter-houses, fishmongers, &c., and the narrowness of the town, favour malignant epidemics. Love affairs alone were in some measure regulated by the *mezouar*. Whoever went about at night without a lantern, was imprisoned. Any Turk or Moor might keep a girl of the town, but he was obliged to state the fact before the *mezouar*. This officer was allowed to punish the girls with blows to the amount of five hundred, or seven hundred. If they were caught in unlawful intercourse with Christians, they were liable to be put into a sack, and thrown into the sea; while the man, to save his life, was obliged to embrace Islamism. Of these girls, the Moorish ones are most apt to be afflicted with syphilis. The French army has two hospitals for its own sick, and there is one for the Moors in the town; at the distance of two English miles from it there is another one for three hundred patients. When we reflect that these hospitals were formerly private residences, and were built almost solely for the purpose of excluding the rays of the sun, it will not be surprising that their arrangement is far inferior to that of a regular French hospital. The number of patients was considerable, the diseases being chiefly dysentery and the typhus fever. Formerly there were also a Spanish and a French hospital, well provided with funds, and every thing required for the treatment of the sick. They were for the benefit of the Spaniards and French who were slaves in Algiers.—*Ib.*

---

*An account of a Fœtus of seven months, with its Placenta adherent to the Nævus occupying the Scalp and Dura Mater.* By ROBERT LEE, M. D., F. R. S.—The fœtus whose malformation forms the subject of the present memoir, was sent to the author by Mr. William Highmore, of Sherborne. Immediately on its arrival in London, a drawing was made of the malformed head, by Mr. Perry, which was presented to the Society in illustration of the description contained in this paper. The vessels of the fœtus and placenta having been minutely injected, the integuments of the head were divided from ear to ear, and the dura mater was found in immediate contact with the skin, all the bones of the vault of the cranium being wanting. The scalp and dura mater on the upper part of the head were almost wholly occupied by a great plexus of dilated arteries and veins, filled with injection resembling nævus. The brain and its immediate envelopes were healthy. The placenta was united to the forehead by a band three-quarters of an inch in breadth, and one and a half inch in length, composed of the amnion and chorion. Into this band the membranes of the brain were protruded through an opening as large as a finger's point. Although the author feels it to be impossible to fix exactly the period when the adhesion of the placenta to the head of the fœtus took place, he thinks it probable that the umbilical cord and band must have been formed about the same time, and at a very early period of the ovum, when the amnion and embryo were in contact, and before the end of the fifth week from the time of conception.—*Ib.*

---

*Combinations of Arsenic and Antimony with Hydrogen. Marsh's apparatus.*—MM. Morh and Leibig have recently published the following observations on Marsh's apparatus for the reduction of arsenic.

By following Mr. Marsh's excellent method for the discovery of minute traces of arsenic, the chemist is liable to fall into error unless he employ the utmost precaution in all cases where the fluid examined contains any other metal. When this occurs the gas disengaged during the process carries over with it some excessively minute drops of the fluid; and these drops contain a portion of the metal, which is reduced by the flame, and becomes attached to the porcelain plate exactly in the same way as the arsenic. It is extremely difficult to condense the drops of fluid alluded to. This cannot be done effectually by passing the gas through a tube twelve inches in length, and filled with fragments of potass; it is better to fill the tube with bits of fine wool; but in every case it is necessary to test the nature of the metallic crust which lines the plate of porcelain. Arsenic is immediately dissolved by nitric acid, the hydrosulphate of potass, &c.

Instead of burning the arseniuretted hydrogen, it might be decomposed by passing it through a tube of one-twelfth of an inch in diameter, heated to redness over a spirit-lamp. The arsenic is thus deposited at about two inches from the orifice of the tube, beyond the red-hot portion, in the form of a brilliant black metallic circle. The other metals which are carried over mechanically by the gas are also reduced, but they remain fixed in the red-hot portion, and are not carried further. The sulphuret of arsenic may be submitted to the same test as arsenious acid and arsenic; or the following method may be employed:—Dissolve the sulphuret in a solution of potass, then drop in a solution of nitrate of silver until the whole of the sulphur be precipitated; next add hydrochloric acid, slightly in excess, filter and precipitate the arsenic acid with lime-water; dry the precipitate carefully, mix it with powdered charcoal and reduce it in the ordinary manner.

On the methods of Marsh and Simon, M. Berzelius remarks, that Mr. Marsh has neglected a property of arseniuretted hydrogen, which may be turned to great account, viz., that of depositing metallic arsenic by heat. The apparatus is extremely simple; we have merely to pass the gas, as it is gradually disengaged, through a tube which is heated to redness over a spirit-lamp; or, if we desire to be more exact, we may place a given quantity of copper (reduced by hydrogen) in the heated portion of the tube, and we thus obtain an arseniuret of copper which will enable us to determine with great precision the quantity of arsenic combined with the hydrogen. To reduce the sulphuret of arsenic M. Simon proposes to employ quick lime instead of the tartrate of lime. M. Berzelius thinks it better to employ light charcoal impregnated with a solution of carbonate of soda, and placed in a tube of 1-24th of an inch in diameter, which is closed at one end. He places the sulphuret first in the tube, and then a few scales of the charcoal, from one-half to an inch in length, and draws out the empty part of the tube to a fine point. He now heats the charcoal over a spirit-lamp, with a double current of air, and then exposes the sulphuret to the flame. The sulphuret passes, at first, into the charcoal unchanged, but as the heat gradually softens the tube, it is decomposed, and the arsenic is sublimed into the pointed end. The same means may be employed for the reduction of arsenic acid.

The ammoniated hydrogen gas discovered by Dr. Thomson contains but a very small quantity of antimony, especially when prepared with zinc and antimony. If the gas thus prepared be disengaged very rapidly, some metallic antimony is attached to the upper part of the matrass, and no change takes place on its being allowed to cool; but if the gas be disengaged very slowly, and allowed to rest, metallic plates of antimony separate after a short time. The arseniuretted hydrogen may be easily distinguished from antimoniated hydrogen by the introduction of a small quantity of chlorine, which precipitates metallic arsenic from the former, but does not produce any precipitate in the latter. The flame of both gases when directed against a porcelain plate, covers it with a metallic crust, but it is easy to distinguish whether the metal be antimony or arsenic, in the following manner:—Dissolve the metallic crust with a few drops of eau regale; then add a few drops of water saturated with sulphuretted hydrogen; this produces a golden-yellow precipitate with arsenic, but an orange-yellow one with antimony; the former is soluble in ammonia, the latter not.—*Jour. Pharm.* Feb. 1839.

---

*On the Venous Circle of the Mammary Areola.* By Professor SEBASTIAN.—In dissecting the mammæ, Professor Sebastian had frequently observed a filament beneath the areola, apparently describing a circle round it; but being unable to procure the gland of a woman giving suck, he for a long while deferred the investigation of its nature. However, by boiling an empty mammæ for twenty-four hours, the close cellular tissue of the organ was so effectually loosened, that an excellent substitute for the full gland was obtained. By examining it he satisfied himself that underneath the skin of the female areola a circle exists, which usually surrounds the greatest part of the base of the nipple at the distance of a line and a half from it. In some cases instead of being circular it is angular, its angles giving origin to branches running towards the circumference of the areola; other smaller twigs ascend from it into the nipple itself. Its vascular and venous nature was proved by injection. The circle exists in the male also, though in him it exhibits a somewhat different form. This anatomical fact has altogether escaped the notice of modern observers, at least no mention is made of it by Meckel, Cloquet, Weber, Lenhossek, &c. The indefatigable Haller, however, distinctly described it in his Elements of Physiology, vol. vii. sect. 1. Sebastian proposes, in consequence, that it be called Haller's circle. As to its use, he believes that it has much to do in the erection of the nipple. Hitherto that part of the breast has been referred to that class of erectile tissues, more on account of its exhibiting the phenomenon of erection, than from anatomical demonstration of its structure. But when the venous circle becomes turgid from being filled with blood, and at the same time the veinules forming communications between it and the nipple are filled, the whole apparatus must push up and cause the erection of the nipple.—*Brit. and For. Med Rev. from Tijdschrift voor Natuurlijke Geschiedenis.* 11 Deel, 3 Stuk.

---

*Influence of Temperature on the Cicatrization of Wounds.* By MM. BRESCHET and GUYOT.—At the sitting of the Academy of Sciences on the 2d July, a joint memoir was presented by these gentlemen, containing the result of some experiments made at the Hôtel Dieu, on the cicatrization of wounds under the influence of an elevated temperature. For this purpose a hot-air bath was contrived in which a diseased limb might be exposed to a dry temperature of 36 centigrade (97° F.) A piece of glass inserted in the top of the apparatus enables the progress of cure to be watched without disturbing the limb. Two cases are detailed, in which, immediately after amputation, the stumps were placed in this apparatus. In the first case in which amputation was performed for scrofulous diseases of the femur, no other dressing was applied than five very narrow strips of emplast. plumib, to approximate the skin without covering the wound, and a roller round the thigh. These slight dressings were removed on the fourth day, when union had taken place, except at the inferior portion: here there was a trifling suppuration, which ceased on the fourteenth day after the removal of the ligatures. The superior three-fourths of the wound remained dry during the whole process of cure. On the fifteenth day there remained a wound from fifteen to twenty lines in length by only one in breadth. The constitutional state of the patient was most satisfactory throughout.

In the second case, that of a man aged sixty-one, amputation was performed on account of suppuration in the ankle-joint. There had been a good deal of constitutional irritation, and the stump was flabby and infiltrated; the tibial artery was ossified. Here union by the first intention was not attempted. Four strips of adhesive plaster were applied, which left a space of eighteen lines between the lips of the wound. For the first five days the wound was covered with a brown scab; on the sixth day suppuration commenced; the appearance of the wound was very favourable; the constitutional symptoms were immediately much ameliorated. This case is not yet terminated; but cicatrization is commencing, and every thing leads to expect a favourable result.—*Ib., from Gazette des Hôpitaux.* July, 1838.

---

*Curious Case of a Spike of Oat introduced into the Bronchi, and expelled through an opening in the walls of the Chest.* By M. Stanski.—A young woman, aged twenty, was brought into the Hôpital Cochin, July 16, 1834. It appeared that she had for some time laboured under consumptive symptoms, and that, a fortnight before her admission, she accidentally swallowed (as she thought) an ear of wild oats, in the act of speaking with it in her mouth: she could not tell which end of the spike went down first. She was immediately seized with a violent fit of choking; which, however, soon went off, and was succeeded by continual convulsive coughing. After two or three days she was attacked with pneumonia of the right lung; and, two or three days subsequently, a sudden fit of coughing was followed by an abundant purulent expectoration of a very fœtid matter, which continued till the time of her admission into the hospital. Upon examination, an abscess was detected in the right lumbar region. This abscess was opened by means of caustic, and twelve ounces of purulent matter, resembling that expectorated, was evacuated. Considerable relief of all the symptoms followed this discharge; but cavernous and amphoric respiration, with pectoriloquy, could be heard at the base of the right lung. Subsequently another abscess formed between the ribs, a little below the inferior angle of the scapula; which, after a month, was also opened with caustic. A seton was introduced into the lower opening of the first abscess, and brought out at the newly-made one: by drawing the cotton upwards, the oat-ear was entangled and brought out, broken into two pieces, which, together, measured three inches in length. After the removal of the foreign body, the wound kept discharging for a considerable time, but ultimately healed, and all the symptoms, both general and auscultatory, decreased; but the frequent pulse and night-sweats remained, and the patient died from phthisis seven months after coming into the hospital.

On examination after death, extensive tubercular disease of the summit of both lungs was found. The lower and back part of the right lung was closely adherent to the ribs by a hard and almost cartilaginous substance: this was continuous with a mass of dense cellular tissue, which descended beneath the pleura costalis, and passed out of the chest between the eleventh and twelfth ribs, close to the outer edge of the sacro-lambalis muscle, and was continued beneath the lumbar fascia, which was separated from the muscles for the space of one and a half or two square inches. The cavity thus formed was lined by a soft, unorganized, false membrane; it did not communicate with the chest, but led by a small fistulous passage to one of the external openings; the ligamentous adhesion was closely applied to the pleura covering the lung, and at that point a small cylindrical canal was found, which communicated with the largest bronchial tube of the inferior lobe of the lung: the canal itself seemed formed by a dilated bronchus. The surrounding substance of the lungs was friable, and of a greyish brown colour; but almost free from tubercles, only one being found.

M. Stanski says that he found as many as twenty cases similar to the preceding, reported in different works, and the effects and symptoms of the accident were always the same. The spikes of different grasses put into the mouth have got into the respiratory tubes, while the patient has been speaking, laughing, or coughing. To get in this way into the trachea, the lower extremity of the spike must have been introduced first otherwise the spikelets would have diverged, and obstructed its passage. It rapidly finds its way into some bronchial tubes, where it excites irritation and suppuration, and eventually reaches the surface of the body, and is discharged along with the matter which surrounds it. An operation for the removal of the ear is to be condemned: it traverses the trachea with the greatest rapidity during the act of inspiration, as is proved by the short duration of the fit of suffocation; and, in all the cases that M. Stanski has found, the process by which the foreign body has been got rid of has never produced death, except when complicated with other disease as in the present case, where the patient evidently died of phthisis after recovering from the accidental affection, and in consequence of tubercles existing in the summit of the lungs previously to the occurrence of the accident.—*Ib. from Gazette Médicale de Paris.* July, 1837.

---

*New Treatment for Bed Sores.*—Dr. Mac Cormick has found the greatest utility from the employment of the following preparation, on the first appearance of any abrasion of the skin, or redness. He coated the parts affected with successive layers of a table varnish, (composed of camphor, spirits of wine, and beeswax,) allowing each layer to dry before applying the next. After five or six layers an artificial cuticle was formed, which always prevented the spread of the inflammation. This remedy originated with Dr. Lendrick, of Sir P. Dun's Hospital, Dublin.—*Lancet.*

